# Gene expression variation in African and European populations of *Drosophila melanogaster*

**DOI:** 10.1186/gb-2008-9-1-r12

**Published:** 2008-01-21

**Authors:** Stephan Hutter, Sarah S Saminadin-Peter, Wolfgang Stephan, John Parsch

**Affiliations:** 1 Section of Evolutionary Biology, Department of Biology, University of Munich, Grosshaderner Strasse, Planegg-Martinsried, 82152, Germany

## Abstract

**Background:**

Differences in levels of gene expression among individuals are an important source of phenotypic variation within populations. Recent microarray studies have revealed that expression variation is abundant in many species, including *Drosophila melanogaster*. However, previous expression surveys in this species generally focused on a small number of laboratory strains established from derived populations. Thus, these studies were not ideal for population genetic analyses.

**Results:**

We surveyed gene expression variation in adult males of 16 *D*. *melanogaster *strains from two natural populations, including an ancestral African population and a derived European population. Levels of expression polymorphism were nearly equal in the two populations, but a higher number of differences was detected when comparing strains between populations. Expression variation was greatest for genes associated with few molecular functions or biological processes, as well as those expressed predominantly in males. Our analysis also identified genes that differed in expression level between the European and African populations, which may be candidates for adaptive regulatory evolution. Genes involved in flight musculature and fatty acid metabolism were over-represented in the list of candidates.

**Conclusion:**

Overall, stabilizing selection appears to be the major force governing gene expression variation within populations. However, positive selection may be responsible for much of the between-population expression divergence. The nature of the genes identified to differ in expression between populations may reveal which traits were important for local adaptation to the European and African environments.

## Abstract 2

A survey of gene expression variation in 16 *Drosophila melanogaster* strains from two natural populations reveals traits that were important for local adaptation to the European and African environments.

## Background

Changes in levels of gene expression can have a large impact on the phenotype of an organism and, thus, provide a rich substrate upon which natural selection can act. Although the importance of gene regulatory changes in adaptive evolution has long been asserted [1], it is only recently that we have begun to uncover the pervasiveness of gene expression polymorphism in natural populations and its role as a source of adaptive variation within species [2-4]. These advances are largely due to the advent of microarray technologies, which allow for the large-scale investigation of differences in transcript abundance among individuals. To date, numerous studies have investigated variation in gene expression in natural populations across a broad range of species, including yeast [5-7], fish [8-10] and hominids [11-14].

The fruit fly *Drosophila melanogaster *has long served as an important model for genetic studies, and is also an important model system for population genetics. Variation at the DNA level in natural populations has been surveyed extensively in microsatellite (for example, [15]) and single nucleotide polymorphism studies (for example, [16,17]). These studies have confirmed that *D. melanogaster *originated from an ancestral population in sub-Saharan Africa and only relatively recently expanded to the rest of the world, a scenario suggested by earlier studies [18,19]. Current populations residing in the ancestral species range show a signal of population size expansion [20,21], while derived populations show the signature of a population bottleneck [16,22]. Extensive theoretical studies have estimated the population genetic parameters associated with these demographic events [23,24].

Most surveys of gene expression variation in *D. melanogaster *have focused on a small number of laboratory strains derived from non-African populations [25-27]. Thus, they do not offer a complete view of expression variation within the species. They are also of only limited value if one wants to detect the effects of demographic events, such as bottlenecks or range expansion, on levels of gene expression variation within natural populations. An exception is the study of Meiklejohn *et al*. [28], which investigated gene expression polymorphism in adult males of eight strains of *D*. *melanogaster*, including four strains from an ancestral population from Zimbabwe and four non-African (cosmopolitan) lab strains. This study uncovered greater levels of variation than previous studies, presumably due to its inclusion of the ancestral African strains. There were, however, some limitations to this work. For example, the sample size was relatively small, with only four African and four non-African strains. Furthermore, the cosmopolitan sample was not from a single population, but instead was a mixture of North American and Asian laboratory stocks. Finally, the Meiklejohn *et al*. study [28] used microarrays designed from an early expressed sequence tag screen of the *D. melanogaster *genome [29] that covered only 42% of the predicted genes.

Here we use whole-genome microarrays to survey gene expression variation in adult males of sixteen strains from two natural populations of *D. melanogaster*, including eight strains from Africa (Zimbabwe) and eight strains from Europe (the Netherlands). DNA sequence polymorphism has already been thoroughly characterized in these two populations [16,20,30]. At the level of gene expression, we find nearly equal amounts of variation within the two populations, but higher amounts in between-population comparisons. Genes associated with a small number of biological processes or molecular functions tend to show higher levels of expression polymorphism than those associated with many processes or functions. These observations suggest that stabilizing selection limits the amount of expression variation within populations. We also find that genes with male-biased expression exhibit higher levels of variation than those with female-biased or unbiased expression, which has implications for the chromosomal distribution of expression-variable genes. Finally, our experimental design allows us to detect genes that differ significantly in expression between the European and African populations, and thus reveals candidates for genes that have undergone adaptive regulatory evolution accompanying the out-of-Africa range expansion of the species.

## Results

### Statistical power

**Figure 1:**
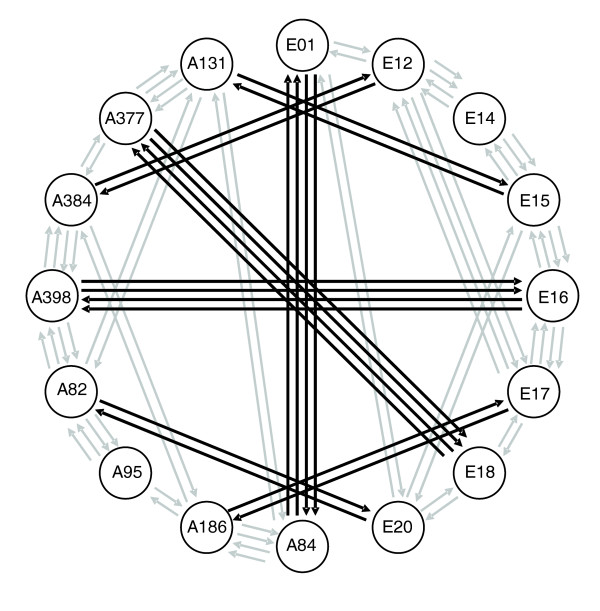
Experimental design. Each circle represents a different *D. melanogaster *strain, with 'E' indicating strains from Europe and 'A' strains from Africa. Gray arrows represent hybridizations performed within populations; black arrows represent hybridizations between populations. Arrows facing in opposite directions represent the dye-swap replicates.

**Figure 2:**
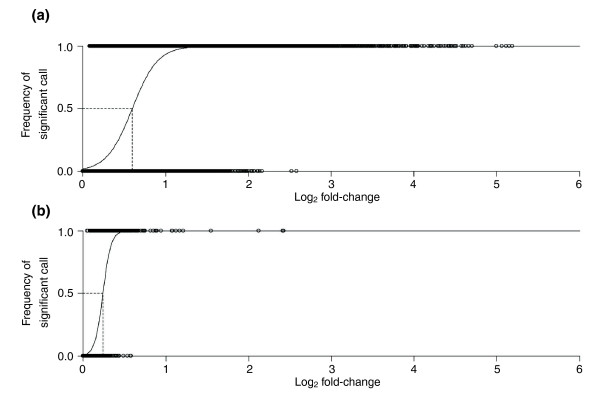
Logistic regression of the probability of detecting significant gene expression differences at the *P *< 0.05 level using BAGEL for **(a) **the quality controlled 16-node experiment and **(b) **the quality controlled 2-node experiment. The dashed line defines the GEL_50 _value on a log_2 _scale.

We performed a total of eighty microarray hybridizations, each of which was a head-to-head comparison of two *D*. *melanogaster *strains (Figure 1). After quality control, 5,048 probes representing 4,512 unique genes had sufficient signal quality to estimate their relative expression level in all 16 strains. This corresponds to approximately 40% of all genes on the array. The complete list of all probes examined in this study is provided as Additional data file 1. The relative expression level of each gene in each strain was estimated using BAGEL (Bayesian Analysis of Gene Expression Levels) [31] and the statistical power of our experiment to detect expression differences between strains was determined by calculating the GEL_50 _statistic [32] (see Materials and methods). The corresponding plot for our data is shown in Figure 2a. The logistic regression reaches a value of 0.5 at a log_2 _fold-change of 0.596, which corresponds to a GEL_50 _of 1.51. In other words, given our experimental design and data quality, there is a 50% chance of detecting a 1.51-fold expression difference as significant at the 5% level. This value compares well with those of similar experiments in fish, yeast, flies, and plants [33], and is slightly better that that of the study of Meiklejohn *et al*. [28] (GEL_50 _= 1.64), which also examined African and non-African *Drosophila*.

We also calculated GEL_50 _values for detecting pairwise differences within or between populations separately. The GEL_50 _was 1.512 within Europe, 1.508 within Africa, and 1.513 between populations, indicating that the power to detect differences in any of these three comparison schemes is approximately equal. This confirms that our experimental design is well balanced and does not have any biases in detecting differential expression within or between populations.

### Total number of differentially expressed genes

Since the number of tests for pairwise differences in expression was extremely high (5,048 probes × 120 pairwise comparisons = 605,760 tests), we could not operate with the conventional 5% significance level due to the problem of multiple testing. We therefore created randomized data sets to estimate the false discovery rate (FDR) at any given significance level (Table 1, 16-node experiment). For all further analyses, we use a *P*-value cut-off of 0.001, which corresponds to a FDR of 6.9% and is similar to the FDR of 5.2% used in the study of Meiklejohn *et al*. [28].

**Table 1:** Number of significant tests and FDRs for different *P*-value cut-offs

	16-node experiment		Two-node experiment	
		
*P*-value	Significant tests	FDR	Significant tests	FDR
0.05	110,285 (18.21%)	0.4906	991 (19.47%)	0.4834
0.02	63,636 (10.51%)	0.3285	562 (11.04%)	0.3292
0.01	44,081 (7.28%)	0.2337	380 (7.47%)	0.2237
0.005	31,670 (5.23%)	0.1657	269 (5.29%)	0.1710
0.002	21,480 (3.55%)	0.1024	161 (3.16%)	0.0870
0.001	16,564 (2.73%)	0.0692	109 (2.14%)	0.0550

Using this cut-off, we found that 1,894 (37.5%) of the probes showed significant differences for at least one pairwise comparison (Table 2), which was slightly lower than the proportion (46.7%) reported by Meiklejohn *et al*. [28]. Since 413 genes were represented by multiple probes in our data set, we checked how well the percentage of polymorphic genes corresponded to the number of polymorphic probes. If a gene was considered polymorphic when at least one of its probes showed a significant pairwise difference between strains, then 38.9% of all expressed genes were polymorphic. If a stricter criterion was applied and only genes for which all probes showed a significant difference were considered polymorphic, this dropped to 35.1%. The overall effect of including multiple probes per gene was rather small. Unless noted otherwise, we present the results on a 'per-probe' basis throughout this paper.

**Table 2:** Expression polymorphism by population

	Polymorphic probes		Mean pairwise differences per probe in %^†^
		
	Total number (%)	Mean per PW (SD)*	
Overall	1,894 (37.5%)	138.0 (53.0)	2.73
Europe	964 (19.1%)	126.5 (43.7)	2.51
Africa	1,039 (20.6%)	125.9 (47.8)	2.49
Between	1,600 (31.7%)	148.4 (57.3)	2.94

*Average number and standard deviation (SD) of probes found to be differentially expressed for each pairwise (PW) comparison between all strains within the corresponding data set.

^†^Average percentage of pairwise comparisons showing differential expression for a probe.

A total of 964 probes (19.1%) showed differences within the European population, 1,039 (20.6%) showed differences within the African population, and 1,600 (31.7%) showed differences when comparing European to African strains (inter-population comparisons). The higher number of differences for the inter-population comparisons was somewhat expected, since there were more pairwise tests than for the within-population comparisons (64 as opposed to 28).

### Expression differences between individual strains

**Figure 3:**
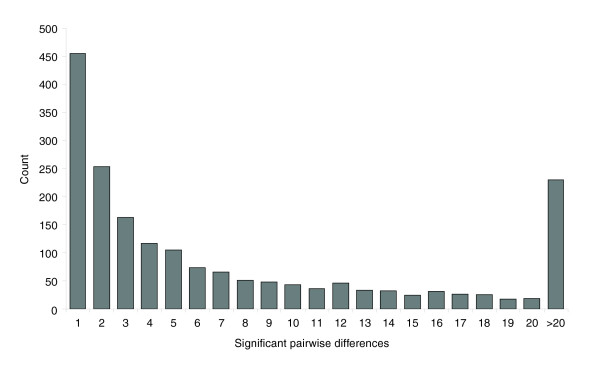
Histogram of the number of significant pairwise differences (*P *< 0.001) for all expressed probes.

We also investigated the number of differentially expressed probes for each pairwise comparison. The complete pairwise comparison matrix is provided as Additional data file 2. On average, 138 probes showed differential expression for each individual pairwise comparison (Table 2). Given the overall number of 1,894 probes that showed differences, this number was surprisingly small, even more so when taking into account that the Meiklejohn *et al*. study [28] detected an average of 498 differentially expressed genes per pairwise comparison with a total number of 2,289 differentially expressed genes. This reveals that, in our data set, there is not much overlap in the lists of differentially expressed genes for the 120 pairwise comparisons. This effect is also visible when comparing the number of pairwise differences detected for each probe. The histogram (Figure 3) shows that a large fraction of probes show significant differences only for 1 or 2 out of the 120 pairwise comparisons.

Expanding this approach to investigate differences within and between populations, we see a pattern resembling that for the total number of differentially expressed probes. On average, comparisons between two European strains showed differences in 126.5 probes, comparisons between two African strains showed differences in 125.9 probes, and comparisons between a European and an African strain showed differences in 148.4 probes (Table 2). Since these numbers are independent from the number of pairwise comparisons, we conclude that there is an excess of differentially expressed probes in the inter-population comparisons (Mann-Whitney *U *test, *P *= 0.019).

To examine expression variation on a gene-by-gene basis, we determined the percentage of significant pairwise differences per probe. In general, this measure of variation followed the pattern seen for the number of differentially expressed genes within the European and African populations presented above (Table 2). The level of expression polymorphism was similar within the African (2.49%) and European (2.51%) populations and a Mann-Whitney *U *test of the two populations was not significant (*P *= 0.086). The between-population comparisons showed a larger proportion of significant tests (2.94%) and this was significantly larger than the within-population polymorphism (Mann-Whitney *U *test, *P *< 0.001).

### The magnitude of expression differences and confirmation by quantitative real-time PCR

**Figure 4:**
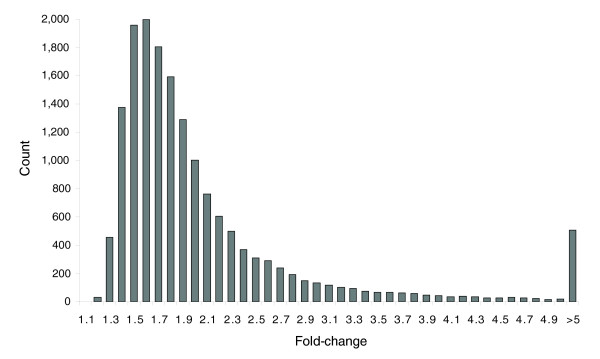
Histogram of the fold-changes in expression for comparisons significant at the *P* < 0.001 level.

**Figure 5:**
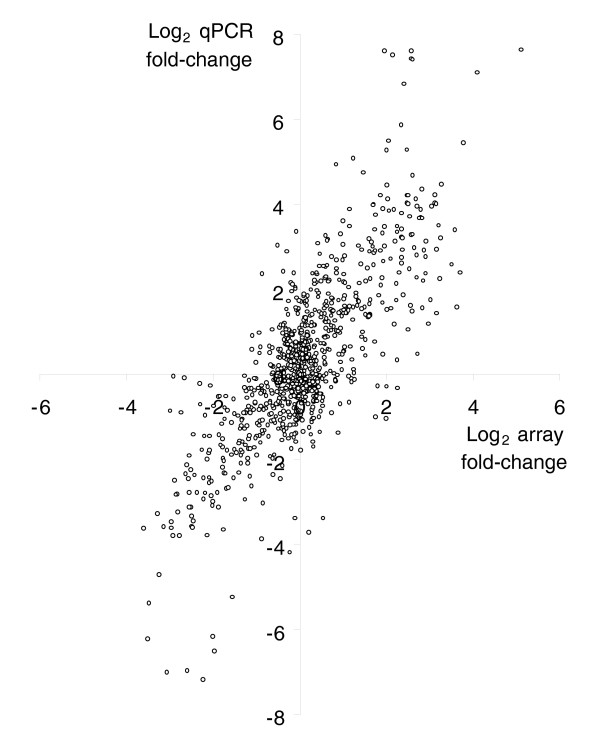
Correlation between fold-change differences in expression measured by microarray and qPCR. Data are from 966 pairwise comparisons of lines across 12 different genes (Pearson's *R* = 0.7, *P* < 0.0001).

In addition to the number of probes that showed differential expression, we also investigated the magnitude of these differences. Of the 605,760 pairwise tests for expression differences, a total of 16,564 were significant at the 0.001 level (Table 1). Figure 4 shows a histogram of the relative fold-changes of these differences. The median fold-change of significant differences was 1.74. The smallest difference that was detected as significant was a fold-change of 1.11, the largest was over 36-fold. As can be seen in Figure 4, the majority of changes were relatively small, falling between 1.2 and 2-fold. To validate the expression differences detected by microarray analysis, we performed quantitative real-time PCR (qPCR) on 12 genes across a total of 966 pairwise comparisons of strains (Additional data file 3). Overall, we observed a strong correlation between the microarray and qPCR results (Figure 5), indicating that the microarrays provide a reliable estimate of the direction and magnitude of gene expression differences between strains.

### Expression polymorphism of X-linked and autosomal genes

We compared the levels of polymorphism for genes residing on the X chromosome to those located on the autosomes and found a systematic difference between these two classes. Levels of expression polymorphism were consistently lower for X-linked genes, irrespective of whether they were measured within or between populations or in the complete data set. Variability on the X chromosome was only about 70% of that on the autosomes when measured as percentage of pairwise differences per probe, and this dearth of polymorphism was statistically significant for all four comparison schemes (Table 3). The same trend was found when using the percentage of polymorphic probes as a statistic, yet the differences between chromosomal classes were not as pronounced (Table 3).

**Table 3:** Expression polymorphism on the X chromosome and autosomes

	X chromosome	Autosomes	X/A ratio*
Number and percentage of polymorphic probes			
Overall	335 (35.8%)	1,559 (37.9%)	0.945 (*P *= 0.22)
Europe	155 (16.5%)	809 (19.7%)	0.838 (*P *= 0.027)
Africa	168 (17.9%)	871 (21.2%)	0.844 (*P *= 0.025)
Between	277 (29.6%)	1,323 (32.2%)	0.919 (*P *= 0.12)
			
Average percentage of pairwise differences			
Overall	2.02	2.90	0.697 (*P *= 0.040)
Europe	1.77	2.68	0.661 (*P *= 0.014)
Africa	1.86	2.64	0.705 (*P *= 0.017)
Between	2.20	3.11	0.708 (*P *= 0.035)

*Deviations from 1:1 expectations for the X/A ratios were tested with two-tailed Fisher's exact tests for the percentage of polymorphic genes and with Mann-Whitney *U *tests for the average number of pairwise differences.

### Expression polymorphism of sex-biased genes

To investigate the contribution of genes with sex-biased expression to overall levels of gene expression variation, we used the consensus results of three independent experiments that directly compared male versus female gene expression in *D*. *melanogaster *[27,34,35] and two different criteria for the classification of sex-biased genes, one based on fold-change and one based on statistical significance [36]. We detected the highest fraction of expressed genes within the male-biased class and the lowest fraction within the female-biased class (Table 4). This is expected, since adult male flies were used as the RNA source for all of our experiments. Meiklejohn *et al*. [28] reported that, when assayed in adult males, genes with male-biased expression were significantly more variable than genes with female-biased or unbiased expression. We observed the same pattern for the genes in our data set: male-biased genes were consistently more variable than genes of the other two classes, and this pattern held for both the European and African populations (Table 4). Female-biased genes tended to have the least expression variation (Table 4). This low variation cannot be explained simply by the lack of expression of the female-biased genes in adult males, because only genes with detectable expression were used in the analysis.

**Table 4:** Expression variation in sex-biased genes

	Two-fold			FDR10%		
		
Sex-bias classification*	Male	Female	Unbiased	Male	Female	Unbiased
Number of genes on array	669	768	3,891	1,228	857	1,534
Percentage of genes detected as expressed	61^†^	22	41	67^†^	33	41
						
Percentage of expressed genes						
Variable in Europe	20^‡^	12	16	22^†^	13	15
Variable in Africa	28^†^	15	16	27^†^	16	17
Variable overall	42^†^	32	31	45^†^	31	32
Differentially expressed between populations	1.21^§^	2.86	3.54	2.46	1.75	3.10
						
Average percentage of pairwise differences						
Within Europe	2.50^†^	1.14	2.07	2.93^†^	1.50	1.82
Within Africa	3.96^†^	1.08	1.75	3.57^†^	1.32	1.75
Overall	3.16^†^	1.09	2.21	3.35^†^	1.50	2.00

*Sex-biased gene sets are defined by Gnad and Parsch [36]. ^†^Significantly different from both female and unbiased (*P *< 0.05) by Fisher's exact test (percentages) or Mann-Whitney *U *test (pairwise differences). ^‡^Significantly different from female (*P *< 0.05) by Fisher's exact test. ^§^Significantly different from unbiased (*P *< 0.05) by Fisher's exact test.

### The effect of gene function on expression polymorphism

**Figure 6:**
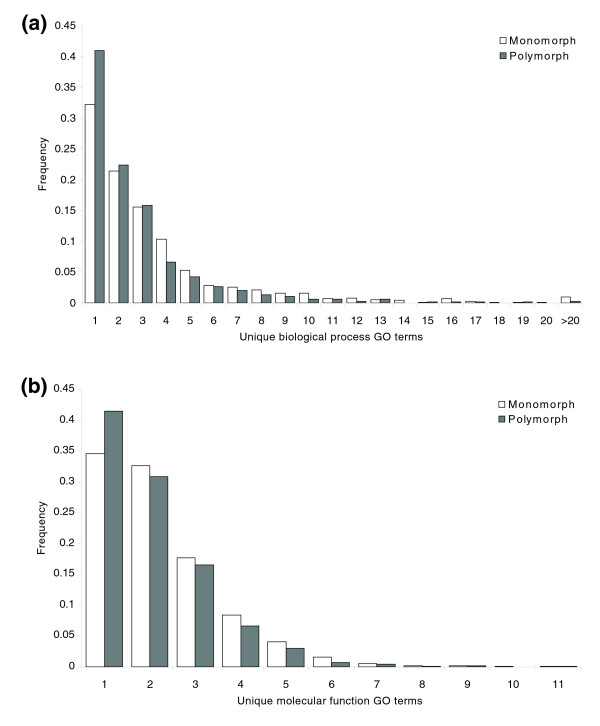
Histogram of the number of unique GO terms associated with monomorphic probes (white) and polymorphic probes (gray). **(a) **GO terms related to biological processes; **(b) **GO terms related to molecular functions.

For a sizable fraction of our data set, the biological processes and/or molecular functions of the genes were (at least partially) known. Of the 5,048 expressed probes, 3,217 were assigned to biological processes, and 3,275 had at least one known molecular function. Some of the probes were associated with more than one Gene Ontology (GO) term, with the extremes being *Egrf *(62 biological processes) and *ninaC *(11 molecular functions). To test whether the number of different processes or functions had an influence on gene expression diversity, we examined the number of GO terms associated with probes that were either polymorphic or monomorphic in expression (Figure 6). There was a relative excess of polymorphic probes associated with a low number of biological processes (three or less) and a paucity associated with four or more processes (Figure 6a). A Mann-Whitney *U *test confirmed that polymorphic probes were associated with fewer GO terms than monomorphic probes (*P *< 0.001). A similar pattern was seen for molecular functions (Figure 6b), where polymorphic probes were associated with fewer molecular functions than monomorphic probes (Mann-Whitney *U *test, *P *< 0.001).

### Expression differences between populations

In order to find genes that differ in expression on a population scale (and are therefore candidates for local adaptation), we pooled all strains of each population into a single node and then used the software BAGEL to find differences between the African and the European nodes (see Materials and methods). With this approach, BAGEL estimates the average expression level for each population and tests for significant differences. Since the polymorphism within a population will affect the variance of this estimate, only those differences will be detected as significant where the within-population variation is small compared to the between-population difference. This new comparison scheme should be much more powerful to detect differences since it has only two nodes to compare with 20 hybridizations. As an additional quality control step, we required that each probe be detected as 'expressed' (see Materials and methods) in at least 9 of the 20 hybridizations. A total of 5,089 probes representing 4,528 genes passed the quality control. The GEL_50 _for this design was 1.18 (Figure 2b), which, as expected, was lower (that is, better) than in the original 16-node analysis.

As with the first analysis, we used a randomized data set to calculate the FDR and adjust our *P*-value for differential expression (Table 1, two-node experiment). We chose a *P*-value cut-off of 0.002, which leads to an FDR of 8.7% and corresponds well to the FDR of the 16-node experiment (6.9%). At this significance level, 161 probes representing 153 genes were differentially expressed between the European and African populations. A complete list of these probes is provided as Additional data file 4. Again, the magnitude of expression differences was relatively low, with the median fold-change difference being 1.32 and the maximum being 5.36. We used qPCR to verify the between-population expression differences for six genes, including two significantly over-expressed in the European population, two significantly over-expressed in the African population, and two with no significant difference between the populations (Table 5). The qPCR results confirmed those of our microarrays for the differentially expressed genes. One of the control genes was detected as having significantly higher expression (at the 5% level) in the African strains by qPCR (Table 5). This may be attributable to increased sensitivity of the qPCR method. However, it should be noted that no multiple-test correction was applied in the qPCR analysis and that this gene is no longer significant after correction for multiple tests.

**Table 5:** Comparison of quantitative PCR and microarray measurements of gene expression differences between populations

Gene	qPCR E/A	Microarray E/A
*Cyp6g1*	3.26 (*P *= 0.0008)	2.12 (*P *< 0.0001)
*CG9509*	0.99 (*P *= 0.003)	1.21 (*P *< 0.0001)
*CG7214*	-2.61 (*P *= 0.002)	-2.42 (*P *< 0.0001)
*CG7203*	-1.57 (*P *= 0.005)	-2.41 (*P *< 0.0001)
*Nap1*	-0.46 (*P *= 0.02)	0.03 (*P *= 0.35)
*CG15295*	0.26 (*P *= 0.60)	0.14 (*P *= 0.17)

Values represent the log_2_ of the mean fold-change difference in expression between the European (E) and African (A) populations as determined by qPCR or microarray. *P*-values for the qPCR were determined by Mann-Whitney *U* tests, and *P*-values for the microarray experiment were determined by BAGEL.

Of the 161 differentially expressed probes, 85 (52.8%) were expressed at a higher level in the African population and 76 (47.2%) were expressed at a higher level in the European population, but this difference was not significant (Fisher's exact test, *P *= 0.26). A comparison on a per-gene basis showed a similar pattern: 80 genes were over-expressed in the African population and 73 in the European population (Fisher's exact test, *P *= 0.25). The magnitude of the expression difference was larger for probes over-expressed in the African population (median fold-change = 1.35) than for probes over-expressed in the European population (median fold-change = 1.27) and this difference was significant (Mann-Whitney *U *test, *P *= 0.044). Neither the X chromosome nor the autosomes were enriched for these probes (Fisher's exact test, *P *= 0.83). There was also no enrichment of sex-biased genes. If anything, sex-biased genes were under-represented among those showing expression differences between the populations (Table 4).

### Functional analysis of candidate genes

Some GO categories were significantly over-represented among the 153 genes with expression differences between populations (Table 6). Furthermore, for some categories the expression differences were biased towards a certain direction. For example, the genes associated with the actin cytoskeleton were all over-expressed in the African population. The GO categories 'actin filament' and 'structural constituent of cytoskeleton' were also exclusively composed of these genes. Interestingly, other genes involved in the formation of *Drosophila *muscles were also over-expressed in the African population, including those encoding myosins, troponins, tropomyosins, and the gene *Zeelin1*. In contrast, we saw the opposite pattern for genes involved in fatty acid metabolism. Here all genes had a higher level of expression in the European population. These genes also form the GO category 'monocarboxylic acid metabolic process' together with the gene *Pgd*, but this gene showed over-expression in the African population. Information on which genes belong to one of the over-represented categories is provided in Additional data file 4.

**Table 6:** GO categories over-represented in the list of genes expressed differentially between populations

GO number	GO term	Genes in genome	Genes in list	*P*-value
Biological process				
GO:0005975	Carbohydrate metabolic process	347	14	7.34E-05
GO:0032787	Monocarboxylic acid metabolic process	48	6	2.24E-05
GO:0006631	Fatty acid metabolic process	38	5	8.67E-05
				
Molecular function				
GO:0016491	Oxidoreductase activity	523	20	4.07E-06
GO:0004448	Isocitrate dehydrogenase activity	4	3	6.83E-06
GO:0005200	Structural constituent of cytoskeleton	12	4	9.34E-06
				
Cellular component				
GO:0015629	Actin cytoskeleton	47	8	7.68E-08
GO:0005884	Actin filament	10	5	5.71E-08

## Discussion

### Patterns of gene expression polymorphism

Our survey of gene expression variation is the largest performed to date in *D. melanogaster *and the first to include a truly natural, derived population. In combination with the ancestral African population, this provides a comprehensive picture of expression variability in the species. However, it should be noted that the amount of expression variation detected among inbred strains may differ from that in natural populations for several reasons. First, inbred strains are expected to be homozygous over a large proportion of the genome and, thus, the effects of dominance on gene expression will not be detected [27]. Second, the process of inbreeding itself may act like an environmental stress and lead to changes in the expression of genes involved in metabolism and stress resistance [37]. Third, mutations that alter levels of gene expression may accumulate in inbred strains during the time that they are maintained in the laboratory [26]. Finally, since all strains were reared in a common laboratory environment, it is not possible to detect genotype-by-environment interactions that affect gene expression. While the above limitations are inherent to this type of microarray study, we expect the general patterns of gene expression polymorphism observed among inbred strains to be robust to these factors and to reflect the patterns present in natural populations.

One pattern we observed was that the amount of expression variation did not differ between the European and the African populations (Table 2). This might seem somewhat surprising, since large-scale genome scans have shown that the African population harbors much more variation (over twice as much) at the DNA level than the European population (for example, [20]), an observation that is consistent with the inferred demographic history of these populations and with the African population having a larger effective size [24,30]. However, the DNA polymorphism studied in such genome scans consists mainly of non-coding single nucleotide polymorphisms, which are thought to evolve (nearly) neutrally. While some authors suggest that differences in gene expression also reflect changes that are selectively neutral [38], more recent studies provide evidence that this is not the case (for example, [39]). Regulatory changes have a direct impact on the phenotype and might affect the fitness of the organism. Most of these changes will have a deleterious effect and the levels of gene expression should, therefore, be under stabilizing selection. Thus, the patterns of expression polymorphism that we observe could be explained by a mutation-selection balance model, where mutations affecting expression level are mostly deleterious and are quickly purged from the population. In such a case, the observable variation depends on the mutation rate and the selection coefficient against deleterious mutations (which should be equal in both of our studied populations), and is independent of the population size [40]. Evidence that stabilizing selection is a key factor governing expression variation has already been found in several studies. For example, mutation accumulation experiments in *Caenorhabditis elegans *[41] and *D. melanogaster *[42] have shown that spontaneous mutations are able to create abundant variation in gene expression. However, when comparing the levels of expression variation in mutation accumulation lines to the levels found in natural isolates, it can be seen that variation in natural populations is significantly lower [41]. Additionally, expression divergence between closely related species was much lower than expected under a neutral model [42]. These results suggest that stabilizing selection plays a dominant role in shaping gene expression variation within species, as well as expression divergence between species.

We observed a higher number of expression differences between populations than within populations, and this result was consistent regardless of the statistic used to quantify expression polymorphism (Table 2). This increased inter-population expression divergence is likely a consequence of population differentiation since the colonization of Europe approximately 16,000 years ago [24,30]. Some of this expression divergence may reflect adaptation to the temperate environment, which would result in genes that show relatively low expression polymorphism within populations, but high expression divergence between populations (discussed below). Nevertheless, the number of genes showing population-specific expression patterns was relatively low compared to overall levels of expression polymorphism. The two-node analysis revealed that only 161 probes had expression levels that were population specific (approximately 3% of all expressed probes). In contrast, 37.5% of all expressed probes showed expression differences between at least two strains in the 16-node experiment. Consequently, distance trees based on gene expression differences had less power to group the strains by population than those based on DNA sequence differences (Additional data file 5).

In both populations, X-linked genes showed consistently less expression polymorphism than autosomal genes (Table 3). This appears to be a result of the unequal genomic distribution of sex-biased genes. Previous studies have shown that male-biased genes are significantly under-represented on the X chromosome [34,35] and also show the highest levels of expression polymorphism [28]. These results are confirmed in our data. Only 9% of the male-biased genes detected as expressed are X-linked; the corresponding proportions for female-biased and unbiased genes are 23% and 17%, respectively. Additionally, we find that male-biased genes show the highest levels of gene expression polymorphism (Table 4). Thus, the reduced expression polymorphism on the X chromosome could be explained by its paucity of male-biased genes. The slight over-abundance of female-biased genes, which show the least expression polymorphism, on the X chromosome may also contribute to this pattern. Indeed, when only genes with unbiased expression are examined, there is no reduction in X-linked expression diversity relative to the autosomes (Additional data file 6).

### Effects of gene function

We examined if functional diversity had any influence on gene expression polymorphism by comparing the number of GO terms associated with monomorphic and polymorphic genes. There are some caveats to this approach. Since GO terms are organized in a hierarchical and network-like fashion, the GO counts do not necessarily correlate in a linear fashion with the functional diversity of a gene. Additionally, the characterization of the gene functions for all genes in the *D. melanogaster *genome is far from being complete. However, these problems should affect both monomorphic and polymorphic genes equally. Thus, we expect any differences between these two groups to have biological relevance.

We find that genes varying in expression among strains tend to be associated with fewer GO terms than those that are monomorphic in expression. This pattern holds for both the number of biological processes and the number of molecular functions that are associated with a gene (Figure 6). A plausible explanation for this is that genes involved in multiple processes or functions are under greater selective constraint to maintain an optimal level of gene expression, because mutations that alter their expression may have deleterious, pleiotropic effects in a greater number of biological pathways. In this respect, our findings mirror previous findings on the relationship between expression variation and number of protein-protein interactions [43,44], which further reinforces our view that stabilizing selection is the dominant force shaping levels of gene expression polymorphism in natural populations.

### Candidate genes for adaptation

To identify genes that are differentially expressed between the European and African populations, we employed a two-node analysis (see Materials and methods), in which all strains from each population were grouped into a single node. An interesting finding was that genes encoding proteins involved in muscle formation were consistently over-expressed in the African population. Two of these genes (*Act88F *and *TpnC41C*) encode proteins that are predominately found in the indirect flight musculature [45,46]. This might be related to differences in the ratio of wing-size/body-size between African and European flies. It is known that *D. melanogaster *populations living close to the equator have smaller wings relative to their body-size than flies inhabiting higher latitudes [47]. It has also been shown that flies that have a small wing area relative to their body size have higher frequencies of wing-beat to overcome the small lift provided by their wings [48]. We therefore hypothesize that the higher expression levels of muscle genes enables African flies to maintain a high-frequency wing-beat. This over-expression of muscle-related genes could be the result of direct selection on their expression, but could also be a downstream effect of selection for increased number or size of muscle cells in African flies. In this context, it is noteworthy that the gene *CG7214*, which has the largest magnitude of over-expression in the African population (5.36-fold), is expressed during wing morphogenesis [49], although its exact function remains unknown. Direct measurements of relative wing sizes, wing-beat frequencies, and number and size of muscle cells in our surveyed populations will provide insight into the phenotypes associated with these gene expression differences. The abundance of highly differentially expressed muscle-related genes in our list might also be the reason why there seems to be more extreme over-expression in the African population.

Genes associated with fatty acid metabolism showed consistent over-expression in the European population. The fat body of *Drosophila *plays an important role in the detoxification of xenobiotics and the defense response to microbial infections and can be viewed as the functional equivalent of the mammalian liver [50,51]. A study comparing the expression profile of dichloro-diphenyl-trichloroethane (DDT)-resistant and DDT-sensitive strains of *D. melanogaster *revealed differences in the expression levels of lipid metabolism genes between these strains [52]. The malic enzyme gene (*Men*), which shows 1.76-fold over-expression in the European population, is of particular interest in this context. This enzyme oxidizes malate to pyruvate and concurrently reduces NADP to NADPH, which is a major reductant in lipid biosynthesis [53]. A study of DNA polymorphism and enzymatic activity of naturally occurring alleles of *Men *revealed clear differences between African and non-African populations [54]. The allelic state of this gene influences not only the abundance of triglycerides in flies, but also the activity of isocitrate dehydrogenase (*Idh*). We find that expression levels of *Idh *also differ between European and African flies (represented by two probes, showing 1.24-fold and 1.18-fold over-expression in European flies), indicating that not only DNA polymorphism, but also variation in expression plays a role in the interaction of these two genes. A classic example of expression differences leading to adaptive phenotypes is the cytochrome P450 gene *Cyp6g1*. It has been shown that over-expression of this gene leads to increased DDT resistance [55]. In our microarray data set, this gene shows the largest magnitude of over-expression in the European population (4.35-fold). We confirmed this pattern by qPCR and found that the actual level of over-expression might even be as high as ten-fold (Table 5). The consistent pattern of higher expression levels in European flies for the above genes provides evidence that the acquisition of resistance against insecticides, such as DDT, is an important adaptive trait for flies living in the European habitat.

## Materials and methods

### Experimental design

Flies were from the European (the Netherlands) and African (Zimbabwe) populations described in Glinka *et al*. [20]. The eight highly inbred strains per population used for the study were randomly chosen. The flies were reared on standard cornmeal-molasses medium at 22°C and a 15 h-9 h light-dark cycle.

The platform used was a genome-wide *D. melanogaster *microarray obtained from the Drosophila Genomics Resource Center (DGRC; Bloomington, IN, USA) known as DGRC-1. This microarray consists of 13,921 exonic PCR amplicons (100-600 bp in length) representing 11,895 unique genes, which is equivalent to 88% of the genome (based on genome annotation 4.1). Since these probes were designed to an earlier annotation of the genome (namely 3.1), some genes are not represented on the array according to updated annotations, while others are represented by more than one probe.

To assess the amount of expression differentiation between any given pair of strains, we developed a hybridization scheme that allowed us to compare all strains while keeping the total number of hybridizations practical (Figure 1). The starting point was a loop design with cross connections that joined strains within each of the two populations (gray arrows in Figure 1). To connect the two loops and allow for comparisons between populations, inter-population hybridizations were performed (black arrows in Figure 1). Each pairwise comparison included a dye swap. This culminated in a total of 30 hybridizations within each population and 20 hybridizations between populations.

### RNA extraction and hybridization

RNA was extracted from 70-75 adult males that were 4-6 days of age using the DGRC protocol [56]. Reverse transcription and labeling were performed with the SuperScript Plus Indirect cDNA Labeling System and Alexa Fluor 555 and 647 dyes (Invitrogen, Carlsbad, CA, USA). RNA from the same extraction was used for the dye-swap replicates. Otherwise, RNA was extracted from a new cohort of flies for each pairwise comparison of strains. Hybridizations were performed following DGRC protocols and arrays were scanned using an aQuire microarray scanner (Genetix, New Milton, UK). All array data have been submitted to the Gene Expression Omnibus database [57] under accession numbers GSM219761-GSM219840 (platform GPL3830, series GSE8843).

### Normalization of raw data

To normalize the signal intensity of the two dye channels for each spot on our arrays, we applied a three-step procedure that is implemented in CARMAweb [58]. This is a web-based interface of the Bioconductor package [59] that provides algorithms to correct for local background effects, within-array variation, and between-array variation. For these corrections, we used the 'minimum', 'printtiploess', and 'quantile' options, respectively. Between-array normalization was performed using the dye-swap replicates for each pairwise comparison of strains.

### Data analysis, quality control and statistical power

The normalized expression ratios for each slide were used as input for BAGEL [31]. This program uses a Markov Chain Monte Carlo algorithm to estimate the relative expression levels of all strains for any given gene. Furthermore, the probability of a gene being differentially expressed between any two strains in the data set is computed.

As a means of quality control, we removed spots that did not show a significant signal of expression, which was determined on a per-slide basis using negative control probes included on the DGRC-1 arrays. Negative controls were defined as the 182 spots on the array consisting of exogenic DNA (for example, genes amplified from yeast or *Escherichia coli*). For each array, the distribution of the signals above background for these negative controls was determined separately for each channel. Subsequently, the signal intensity in each channel for each spot representing a gene was compared to the negative distribution. If the signal of a spot fell within the upper 5% of the negative distribution in each channel, the gene was considered 'expressed'. If a spot presented a signal that was lower than this threshold in either of the two channels, then it was considered 'non-expressed' and was excluded from further analysis.

To determine the experiment-wide FDR, we repeated the BAGEL analysis on a randomized version of our final data set. Randomization was performed on the input file by sampling with replacement within each hybridization (that is, randomizing within a column), thereby maintaining the underlying data structure (for example, missing data) within each hybridization. Random sampling was carried out until a total of 5,048 randomized probes were generated, which corresponds to the total number of expressed probes in the original data set. This allowed for an easy and direct comparison of observed and randomized data.

To estimate the power of our experiment to detect expression differences between strains, we calculated the GEL_50 _statistic, which has been proposed as a standard measure to compare studies of expression variation across different experiments and platforms [32]. The GEL_50 _is defined as the expression difference at which there is a 50% chance of detecting significance at the 5% level. To obtain this statistic, all pairwise comparisons of differential gene expression are assigned a value of one if they are significant or zero if they are non-significant. These zeros and ones are then plotted on a graph as a function of the expression difference (that is, the fold-change) between the two samples (on a log_2 _scale). Afterwards, a logistic function is fitted through the data points and the GEL_50 _is defined as the fold-change at which the logistic function reaches 0.5.

### Detection of differentially expressed genes between populations

To identify genes that differ in expression between the African and the European populations, we repeated the BAGEL analysis using only hybridizations in which an African strain was compared directly to a European strain. This resulted in a total of 20 hybridizations (black arrows in Figure 1). All African strains were combined into a single node named 'Africa' and all European strains where combined into a node named 'Europe'. With this approach, the different strains used within each population can be considered as biological replicates. To determine the FDR, a randomized data set was created by permuting the expression ratios of the replicate hybridizations within each gene (that is, randomizing within a row). This has the effect of randomly assigning the ratio of each hybridization as either Europe/Africa or Africa/Europe. It ensures that the proportion of missing data remains constant in the overall data set as well as within each gene, leading to equal distributions of missing data per gene in the observed and the randomized data sets. Furthermore, the randomized data set automatically contained 5,089 randomized probes that could be directly compared to the observed data. Additionally, we created a randomized data set using the approach of the 16-node experiment (see above) for comparison. Both methods produced very similar results (data not shown) and the first approach was used for our analysis.

### Quantitative real-time PCR

To validate the gene expression differences between strains and populations detected by our microarray analyses, we performed quantitative real-time PCR on a subset of genes. This included genes that showed a high number of significant expression differences within Europe (*CG18180 *and *CG8997*), within Africa (*CG15281 *and *CG5791*), or in the combined sample (*Cyp6a2 *and *CG18179*). In addition, we included genes showing significant expression differences between the two populations, including two with higher expression in Europe (*Cyp6g1 *and *CG9509*) and two with higher expression in Africa (*CG7214 *and *CG7203*). Finally, we included two control genes that did not show any significant expression differences within or between populations (*Nap1 *and *CG15295*).

Prior to qPCR, 5 μg of total RNA was reverse transcribed using Superscript II reverse transcriptase (Invitrogen) and random hexamer primers. The resulting cDNA was used at 1:40 dilution for qPCR using TaqMan probes and a 7500 Fast Real-Time PCR System (Applied Biosciences, Foster City, CA, USA). The probe IDs for the target genes (in the order listed above) were as follows: Dm01801887_s1, Dm01791303_g1, Dm01791414_s1, Dm02147133_g1, Dm01817955_g1, Dm01801878_s1, Dm01819889_g1, Dm01838873_g1, Dm02365366_s1, Dm01809356_g1, Dm01842610_g1, and Dm02539051_s1. Three replicate assays were performed for each sample and the threshold cycle value (Ct) was averaged across these replicates. Expression levels of the target genes were standardized using the ribosomal protein gene *RpL32 *(Dm02151827_g1) as an endogenous control. For this, a ΔCt value was calculated by subtracting the control Ct value from the target Ct value. The fold-change, which represents the difference in expression between two samples (ΔCt_1 _and ΔCt_2_), was calculated as 2^-(Δ*Ct*_1_-Δ*Ct*_2_)^. For comparisons between the European and African populations, ΔCt values were averaged within each population and the African value was used as ΔCt_2 _for fold-change calculation.

### Gene ontology

A list of all GO terms describing molecular functions and biological processes associated with the probes on the microarrays was downloaded directly from the DGRC website [56]. Of the 13,921 probes representing a gene, at least one biological process was known for 8,251 and at least one molecular function was annotated for 8,523. We calculated the number of unique GO terms describing molecular functions and biological processes associated with each probe to get an estimate of its 'functional diversity'.

To test if a functional category was over-represented in our list of differentially expressed genes, we used the web-based tool g:Profiler [60]. This tool introduces a new correction for multiple testing (called g:SCS) that takes the hierarchical nature of GO terms into account.

## Abbreviations

BAGEL, Bayesian Analysis of Gene Expression Levels; DDT, dichloro-diphenyl-trichloroethane; DGRC, Drosophila Genomics Resource Center; FDR, false discovery rate; GO, gene ontology; qPCR, quantitative real-time PCR.

## Authors' contributions

The study was conceived and designed by SH, SSS, WS and JP. The microarray experiments were performed by SH and SSS, and the quantitative real-time PCR experiments were carried out by SSS. The microarray data were analyzed by SH and JP, and the quantitative real-time PCR data were analyzed by SSS. The manuscript was written by SH, SSS and JP with input from WS. All authors have read and approved the final manuscript.

## Additional data files

The following additional data are available. Additional data file 1 is a table listing all probes that were found to be expressed in the 16-node experiment along with the relative expression levels for each strain and the *P*-values for each pairwise comparison as calculated by BAGEL. Additional data file 2 is a table showing the number of differentially expressed probes for each of the 120 pairwise comparisons between strains at the *P *< 0.001 significance level, as well as the random expectations. Additional data file 3 is a table showing the ΔCt values obtained by qPCR for the 12 genes surveyed. Additional data file 4 is a table listing all probes showing differential expression between Europe and Africa on a population level as detected by the two-node experiment, including relative expression levels, *P*-values and over-represented GO categories. Additional data file 5 is a figure comparing neighbor-joining trees of the 16 strains created using DNA polymorphism data and the gene expression distance matrix. Additional data file 6 is a table comparing the variability of non sex-biased genes between the X chromosome and the autosomes.

## Supplementary Material

Additional data file 1 Probes expressed in the 16-node experiment and the relative expression levels for each strain and the *P*-values for each pairwise comparison as calculated by BAGEL. Click here for file

Additional data file 2 Number of differentially expressed probes for each of the 120 pairwise comparisons between strains at the *P* < 0.001 significance level, as well as the random expectations. Click here for file

Additional data file 3 ΔCt values obtained by qPCR for the 12 genes surveyed. Click here for file

Additional data file 4 The table includes relative expression levels, *P*-values and over-represented GO categories. Click here for file

Additional data file 5 Comparison of the neighbor-joining trees of the 16 strains created using DNA polymorphism data and the gene expression distance matrix. Click here for file

Additional data file 6 Comparison of the variability of non sex-biased genes between the X chromosome and the autosomes. Click here for file
