# Supplementary material for: Gene expression variation in African and European populations of Drosophila melanogaster
Source: Genome Biol. 2008 Jan 21;9(1):R12. doi: 10.1186/gb-2008-9-1-r12 (PMC2395247; doi:10.1186/gb-2008-9-1-r12)
Supplement: Additional data file 2 — Number of differentially expressed probes for each of the 120 pairwise comparisons between strains at the P < 0.001 significance level, as well as the random expectations. [file gb-2008-9-1-r12-S2.doc]

|  | *E01* | *E12* | *E14* | *E15* | *E16* | *E17* | *E18* | *E20* | *A82* | *A84* | *A95* | *A131* | *A186* | *A377* | *A384* | *A398* |
| --- | --- | --- | --- | --- | --- | --- | --- | --- | --- | --- | --- | --- | --- | --- | --- | --- |
| *E01* |  | 9 | 2 | 7 | 6 | 11 | 7 | 6 | 10 | 9 | 13 | 8 | 17 | 12 | 14 | 5 |
| *E12* | 168 |  | 5 | 10 | 7 | 7 | 4 | 8 | 18 | 12 | 14 | 13 | 7 | 10 | 16 | 11 |
| *E14* | 74 | 151 |  | 8 | 7 | 3 | 4 | 2 | 3 | 10 | 9 | 9 | 6 | 8 | 3 | 5 |
| *E15* | 93 | 145 | 137 |  | 8 | 6 | 7 | 9 | 9 | 6 | 14 | 19 | 11 | 8 | 7 | 11 |
| *E16* | 99 | 111 | 92 | 76 |  | 4 | 3 | 7 | 9 | 9 | 13 | 7 | 5 | 5 | 10 | 16 |
| *E17* | 80 | 255 | 114 | 151 | 221 |  | 5 | 4 | 12 | 9 | 15 | 6 | 6 | 6 | 9 | 10 |
| *E18* | 91 | 99 | 92 | 96 | 98 | 94 |  | 5 | 9 | 7 | 12 | 4 | 6 | 8 | 5 | 5 |
| *E20* | 139 | 156 | 106 | 174 | 145 | 117 | 168 |  | 9 | 19 | 25 | 9 | 12 | 10 | 8 | 16 |
| *A82* | 131 | 164 | 109 | 92 | 142 | 148 | 104 | 280 |  | 16 | 25 | 20 | 11 | 11 | 9 | 7 |
| *A84* | 180 | 132 | 108 | 79 | 97 | 110 | 79 | 154 | 72 |  | 23 | 10 | 15 | 10 | 14 | 9 |
| *A95* | 216 | 220 | 153 | 112 | 168 | 299 | 165 | 322 | 180 | 127 |  | 19 | 23 | 13 | 13 | 13 |
| *A131* | 109 | 121 | 95 | 42 | 98 | 98 | 129 | 150 | 133 | 80 | 188 |  | 8 | 6 | 12 | 16 |
| *A186* | 118 | 147 | 93 | 83 | 110 | 52 | 105 | 165 | 89 | 167 | 192 | 105 |  | 6 | 6 | 8 |
| *A377* | 126 | 180 | 131 | 105 | 139 | 120 | 229 | 188 | 97 | 78 | 178 | 116 | 88 |  | 7 | 4 |
| *A384* | 128 | 228 | 123 | 135 | 197 | 160 | 148 | 187 | 148 | 112 | 240 | 105 | 157 | 102 |  | 4 |
| *A398* | 180 | 222 | 161 | 145 | 275 | 157 | 110 | 245 | 54 | 66 | 200 | 93 | 109 | 84 | 164 |  |

Numbers below the diagonal are the observed differences; numbers above the diagonal are from a randomization of the data (*i.e*., the expected number of false positives)
