# Supplementary material for: Gene expression variation in African and European populations of Drosophila melanogaster
Source: Genome Biol. 2008 Jan 21;9(1):R12. doi: 10.1186/gb-2008-9-1-r12 (PMC2395247; doi:10.1186/gb-2008-9-1-r12)
Supplement: Additional data file 3 — ΔCt values obtained by qPCR for the 12 genes surveyed. [file gb-2008-9-1-r12-S3.doc]

| Gene | European strains | | |  |  |  |  |  | African strains | |  |  | |  | |  | |  | |  | |
| --- | --- | --- | --- | --- | --- | --- | --- | --- | --- | --- | --- | --- | --- | --- | --- | --- | --- | --- | --- | --- | --- |
|  | *E01* | *E12* | *E14* | *E15* | *E16* | *E17* | *E18* | *E20* | *A82* | *A84* | *A95* | | *A131* | | *A186* | | *A377* | | *A384* | | *A398* |
| *Cyp6g1* | -2.394 | -2.773 | -2.564 | -2.973 | -2.886 | -2.355 | -2.794 | -2.976 | 0.429 | 4.622 | 1.229 | | -0.625 | | -1.9 | | -1.554 | | 1.233 | | 0.894 |
| *CG7214* | 10.616 | 10.247 | 9.685 | 7.927 | 8.635 | 9.829 | 10.028 | 11.583 | 4.758 | 7.641 | 8.239 | | 7.142 | | 7.782 | | 6.907 | | 8.111 | | 7.119 |
| *CG7203* | 3.694 | 2.883 | 3.582 | 3.059 | 1.783 | 3.163 | 2.496 | 4.248 | 1.912 | 1.312 | 1.453 | | 1.324 | | 0.408 | | 2.789 | | 0.305 | | 2.828 |
| *CG9509* | 2.983 | 2.295 | 3.063 | 2.464 | 2.394 | 3.768 | 2.495 | 2.611 | 4.041 | 3.367 | 3.826 | | 3.936 | | 2.93 | | 4.066 | | 3.894 | | 3.899 |
| *Cyp6a2* | 5.893 | 3.447 |  | 3.029 | 2.796 | 2.414 | 1.539 | 2.758 |  | 4.226 | 5.433 | |  | | 3.331 | | 2.687 | |  | | 4.553 |
| *CG18179* |  |  | 1.739 |  |  | 4.647 |  | 9.366 | 7.014 | 3.132 |  | | 3.982 | |  | |  | | 2.272 | |  |
| *CG5791* | 3.927 |  |  |  | 4.585 | 3.733 | 3.428 |  | 4.392 | 5.102 |  | | 4.437 | |  | |  | |  | |  |
| *CG15281* |  |  | 5.286 |  | 8.427 | 7.019 | 8.893 | 7.465 | 21.482* | 15.985* | 7.848 | |  | | 8.544 | | 6.709 | |  | | 7.505 |
| *CG8997* | 1.202 |  | 1.739 |  | 1.957 | 0.067 | 2.654 |  | -1.336 | -1.092 |  | | 0.87 | |  | | 0.817 | | 1.028 | | 0.454 |
| *CG18180* |  |  | -0.306 |  | 2.18 | 1.458 |  | 5.125 | 3.664 | 1.51 |  | |  | |  | | 2.601 | |  | | 2.69 |
| *CG15295* | 10.775 | 10.643 | 11.401 | 10.726 | 11.499 | 11.166 | 11.344 | 11.801 | 10.749 | 12.758 | 11.164 | | 11.685 | | 11.048 | | 11.676 | | 11.034 | | 11.338 |
| *Nap1* | 3.304 | 3.167 | 3.407 | 3.182 | 3.021 | 2.752 | 2.703 | 3.174 | 1.458 | 2.598 | 3.003 | | 2.722 | | 2.443 | | 2.991 | | 3.068 | | 2.779 |

* Expression ratios for several comparisons involving these strains (which have very low expression levels for *CG15281*) fell outside of the axis boundaries of Figure 5 and are, therefore, not shown. However, all data points were included in the regression analysis.

Empty cells indicate that expression levels of the gene were not measured for the corresponding strain.
