# Supplementary material for: Gene expression variation in African and European populations of Drosophila melanogaster
Source: Genome Biol. 2008 Jan 21;9(1):R12. doi: 10.1186/gb-2008-9-1-r12 (PMC2395247; doi:10.1186/gb-2008-9-1-r12)
Supplement: Additional data file 5 — Comparison of the neighbor-joining trees of the 16 strains created using DNA polymorphism data and the gene expression distance matrix. [file gb-2008-9-1-r12-S5.doc]

A

B

Neighbor-joining trees of the 16 *D*. *melanogaster* strains based on (a) DNA polymorphism data from the study of Ometto *et al.* [16] and (b) the gene expression distance matrix provided as Additional data file 2. Trees were created using the PHYLIP software package [61]. Bootstrap values for the gene expression tree were generated by sampling with replacement of the gene list and are given as percentages for nodes with a support of >50%. As expected for neutral DNA data from two semi-isolated, random-mating populations, the tree based on non-coding SNPs forms two distinct star-like clades separating the African and the European strains (in red and blue, respectively). Note the increased length of the African external branches indicating elevated polymorphism in Africa. For the tree based on expression differences, strains from the same population do not form monophyletic clades. This is due to the small number of genes showing distinct population specific expression relative to overall levels of polymorphism. Nevertheless, strains originating from the same population tend to cluster together, even though bootstrap support for internal nodes is low.
