# Supplementary material for: Gene expression variation in African and European populations of Drosophila melanogaster
Source: Genome Biol. 2008 Jan 21;9(1):R12. doi: 10.1186/gb-2008-9-1-r12 (PMC2395247; doi:10.1186/gb-2008-9-1-r12)
Supplement: Additional data file 6 — Comparison of the variability of non sex-biased genes between the X chromosome and the autosomes. [file gb-2008-9-1-r12-S6.doc]

| Sex-bias classification^a^ |  | 2-fold |  |  |  | FDR10% |  |
| --- | --- | --- | --- | --- | --- | --- | --- |
|  | X | autosomes | X:A ratio |  | X | Autosomes | X:A ratio |
| Number of unbiased genes | 262 | 3629 | – |  | 108 | 1426 | – |
| *Percentage of expressed genes* | | | | | | | |
| Variable in Europe | 14 | 16 | 0.86 |  | 22 | 14 | 1.61^b^ |
| Variable in Africa | 19 | 16 | 1.23 |  | 20 | 16 | 1.24 |
| Variable overall | 32 | 31 | 1.04 |  | 39 | 30 | 1.28 |
| *Average percentage of pairwise differences* | | | | | | | |
| Within Europe | 1.57 | 2.18 | 0.71 |  | 1.75 | 1.82 | 0.96 |
| Within Africa | 1.82 | 1.71 | 1.06 |  | 1.18 | 1.89 | 0.63 |
| Overall | 1.93 | 2.26 | 0.86 |  | 1.59 | 2.08 | 0.76 |

^a^ Sex-biased gene sets are defined by Gnad and Parsch [36].

^b^ Significantly different from 1:1 (*P* = 0.04) by Fisher's exact test. All other comparisons are not significant.
